# A team without a name: emergency medicine recognition and its impact on working conditions and well-being

**DOI:** 10.1007/s00063-025-01275-8

**Published:** 2025-05-02

**Authors:** Megan Gates Kemnitz, Eugenia-Maria Lupan-Muresan, Francis Somville, Bruno Barcella, Noaa Shopen, María de los Angeles López Hernández, Eric P. Heymann

**Affiliations:** 1 Zentrale Notaufnahme, Diakonie Klinikum Stuttgart, Stuttgart, Germany; 2 Emergency Department, Durham Veterans Affairs Medical Center, Durham, NC USA; 3 Emergency Medicine Discipline, Department 6—Surgery, “Iuliu Hatieganu” University of Medicine and Pharmacy Cluj-Napoca, Cluj-Napoca, Romania; 4 Emergency Department, County Emergency Hospital Cluj-Napoca, 3–5 Clinicilor Street, 400006 Cluj, Romania; 5 Department of Emergency Medicine, University of Antwerp, UZA, Edegem, Belgium; 6 Department of Emergency Medicine, Ziekenhuis Geel, Geel, Belgium; 7 Faculty of Medicine and Health Sciences, University of Antwerp, Wilrijk, Belgium; 8 Faculty of Medicine, University of Leuven, Leuven, Belgium; 9 Experimental Medicine PhD Program, University of Pavia, Pavia, Italy; 10 Emergency Medicine Unit, IRCCS Policlinico San Matteo, Pavia, Italy; 11 Emergency Medicine Department, Tel Aviv Medical Center, Tel Aviv, Israel; 12 Faculty for Medicine, Tel Aviv University, Tel Aviv, Israel; 13 Emergency Medicine, Hospital Universitario de Canarias, Tenerife, Islas Canarias Spain; 14 Department of Emergency Medicine, Cantonal Hospital of Neuchâtel, Neuchâtel, Switzerland; 15 Department of Emergency Medicine, Inselspital, Bern University Hospital, University of Bern, Bern, Switzerland

**Keywords:** Medical specialty, Emergency medical services, Primary care, Psychosocial risk, Empowerment, Medizinische Fachrichtung, Medizinische Notfallversorgung, Grundversorgung, Psychosoziale Gefährdung, Befähigung

## Abstract

Emergency medicine (EM) has evolved significantly over the past 50 years, transitioning from a focus on acute injuries and illnesses to include primary and specialty care, disaster response, and social issues. To date, nearly 60 countries have officially recognized EM as a medical specialty. However, growing patient demands, healthcare staff shortages, and an aging population have strained emergency departments, worsening working conditions for EM professionals and compromising patient care. To address these challenges, formal recognition of EM as a specialty is crucial.

As a specialty, EM offers significant benefits. It improves patient outcomes by ensuring structured, standardized training that equips specialists with the skills to manage acute conditions such as trauma, stroke, and myocardial infarction. Countries with recognized EM specialties have reported reduced morbidity and mortality and enhanced healthcare resilience during crises like pandemics and mass casualty events. Additionally, professional recognition aids in recruitment, retention, and reducing burnout among EM practitioners by establishing clear career pathways. Furthermore, it ensures specific paraclinical training in areas such as patient flow, and it strengthens healthcare systems. However, despite these benefits, challenges remain. Resource diversion from primary care, increased healthcare costs, and the initial investment required for training programs are potential drawbacks to EM specialty recognition. Achieving EM recognition will require a strategic collaborative approach, focusing on education, professional support, and collaboration across healthcare sectors.

**Supplementary Information:**

The online version of this article (10.1007/s00063-025-01275-8) includes Appendix A.

## Abstract (de)

Die Notfallmedizin hat sich in den letzten 50 Jahren deutlich weiterentwickelt – mit Verlagerung des bisherigen Fokus auf akute Verletzungen und Erkrankungen zur aktuellen Einbeziehung von Primär- und spezialisierter Versorgung, des Katastrophenschutzes und sozialer Probleme. Steigende Patientenzahlen, Personalmangel im Gesundheitswesen und eine alternde Bevölkerung führen jedoch zu einer Überlastung der Notaufnahmen, was die Arbeitsbedingungen für das Notfallpersonal verschlechtert und die Patientenversorgung beeinträchtigt. Um diese Herausforderungen zu bewältigen, ist die formale Anerkennung der Notfallmedizin als Fachgebiet, wie sie heute in fast 60 Ländern erfolgt ist, entscheidend. Als Fachgebiet bietet die Notfallmedizin Vorteile. So werden die Ergebnisse für die Patienten durch strukturierte, standardisierte Ausbildung verbessert, welche die Experten mit den Fähigkeiten ausstattet, akute Zustände wie Trauma, Schlaganfall und Myokardinfarkt zu versorgen. In Ländern mit Notfallmedizin als anerkanntem Fachgebiet wurde über eine verminderte Morbidität und Mortalität sowie verstärkte Resilienz im Gesundheitswesen während Krisen wie Pandemien und Massenanfall von Verletzten dokumentiert. Allerdings bleiben trotz dieser Vorteile Herausforderungen bestehen. Die Ressourcenumleitung von der Primärversorgung, gestiegene Gesundheitsversorgungskosten und die anfängliche Investition, die für die Ausbildungsprogramme erforderlich ist, sind potenzielle Hindernisse für die Anerkennung der Notfallmedizin als Fachgebiet. Das Erreichen der Anerkennung der Notfallmedizin als Fachgebiet erfordert einen strategischen, kooperativen Ansatz mit dem Fokus auf Ausbildung, professionelle Unterstützung und gesundheitssektorübergreifende Zusammenarbeit.

## Introduction and background

Emergency medicine (EM) has grown rapidly in the past 50 years. From its initial calling of treating acute and critical injuries and illnesses, its role has expanded, and emergency departments (ED) now often serve as the first port of call for primary and specialty care, in addition to significant roles in prehospital and disaster medicine as well as social-societal issues (homelessness, addiction, geriatric care).

**Fig. 1:**
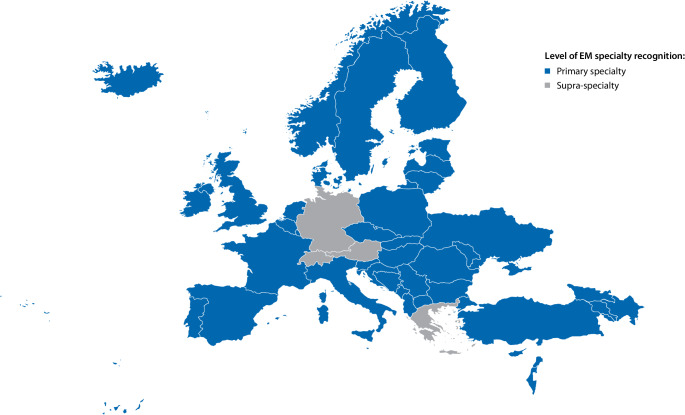
Recognition of emergency medicine (*EM*) in Europe. (Source: European Society of Emergency Medicine)

Acknowledging the unique training and skills required to effectively fill these expanding roles, nearly 60 countries have officially recognized EM as a medical specialty (see Fig. 1; [1]).

**Fig. 2:**
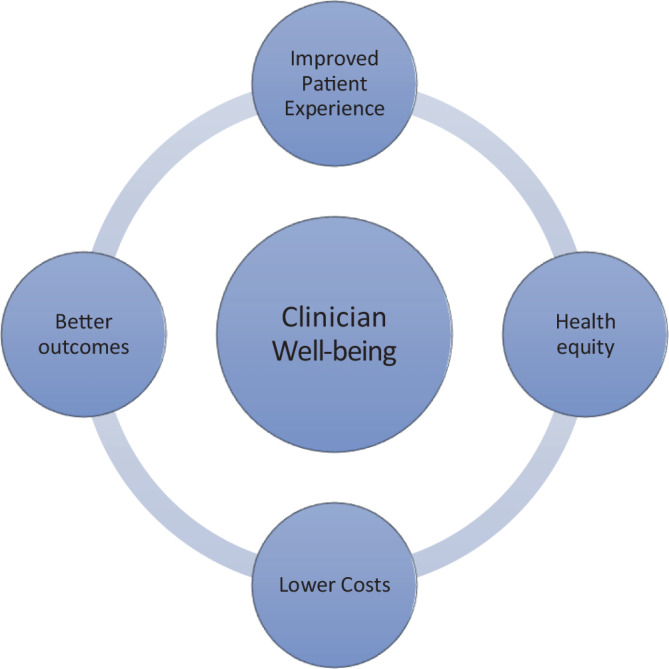
Sick clinicians cannot treat sick patients: the Quintuple Aim

The current decreased primary care and specialty accessibility and an aging population have resulted in a continuous increase in the number of consultations to EDs worldwide [2, 3]. These challenges have been compounded by a decline in the supply of healthcare provision relative to demand [4]. As a result of these increases, which have not been matched by investments, working conditions have deteriorated for EM professionals, compromising the quintuple aim of patient experience, population health, reducing costs, clinician well-being, and health equity (see Fig. 2). Today, EM has reached a crisis point, globally. To ensure its perennity, and maintain healthcare security for patients worldwide, reform is urgently required.

In order to improve working conditions and well-being in EM, it is time for global recognition of the specialty. Learning from experiences in countries where this has already been established, this paper delves into the benefits and difficulties associated with developing the discipline. To do this, we review commonalities between eight countries that have gone through the process (United States, Romania, Belgium, Italy, Israel, the Netherlands, Portugal, and Spain), and use this as an opportunity to dissipate myths and misconceptions about what EM as a specialty means (see Appendix A in the online supplementary information).

## Benefits of recognizing emergency medicine as a specialty

Establishing the specialty of EM brings significant benefits to patient care, the healthcare system, and clinician well-being.

### Benefits for patient care and outcomes

Adapting to the increase in patient volume and complexity requires structured and standardized training pathways to equip healthcare providers with the skills necessary for efficient, high-quality emergency care. Training specific to EM prepares specialists to make rapid, precise diagnoses and deliver timely, evidence-based interventions with the goal of minimizing diagnostic errors and enhancing outcomes [5]. This is important for all conditions, especially in critical time-sensitive pathologies such as myocardial infarction, stroke, or trauma. Optimizing the initial evaluation and management of acutely ill patients improves the efficiency and quality of care, a finding confirmed by studies showing that countries with recognized EM specialties have observed reductions in systemic morbidity and mortality [6]. The Advanced Trauma Life Support (ATLS) approach illustrates the value of standardized training ameliorated by emergency physicians (EPs). Initially developed by an orthopedic surgeon in 1976, ATLS provides a specific, systematic, protocol-driven approach to trauma. It has been widely adopted by EPs, who have participated in the further development of the program [7], and has led to decreased trauma mortality rates [8]. Similar examples include stroke management (and door-to-lysis delay improvement; [9]), acute coronary syndrome [10, 11], and sepsis management [12], where EPs have built upon and enacted protocols developed by other specialties, to improve the time-sensitive response to the emergency component of each algorithm. The formalization of EM as a specialty further supports collaboration with other specialties as well as the development and adoption of evidence-based protocols and consistent care standards, enhancing the quality and reliability of emergency care across healthcare systems, independent of location.

In addition to their technical expertise, EM practitioners play a crucial role in coordinating care for complex cases. Emergency physicians facilitate timely referrals to appropriate specialists, ensuring smooth transitions and continuity of care beyond the ED. Their training emphasizes not only clinical skills but also patient-centered communication, empathy, and culturally sensitive care, which are essential in managing patients from diverse backgrounds in high-stress settings. Additional skills in leadership and management, particularly in settings of patient surge and backlog, have been identified as promoters of facilitated patient flow and are a focus of ongoing curriculum development for EPs [13].

These foundational elements were key arguments for the formal establishment of EM as a specialty in countries such as the United States, Romania, Belgium, Italy, Israel, the Netherlands, Portugal, and Spain (see Infobox).

#### Infobox

“From an objective point of view, the specialty of emergency medicine (EMUE in Spanish) means many issues: homogeneous, regulated, structured training for professionals; therefore, the best possible guarantee of quality and safety in care. It means recognition of a specialty that already existed ‘de facto’ but not ‘registered’; and the latter has repercussions at the European level (traffic of professionals). It means equity in care, by guaranteeing maximum competence for the care of time-dependent pathologies”.

*Dr. Vázquez Lima, president of the ****Spanish Society of Urgent and Emergent Care*** *(Sociedad Española de Medicina de Urgencias y Emergencias) in response to the acknowledgement of the specialty of Emergency and Urgent Care Medicine in September, 2024* [14].

### Benefits for the healthcare system

A well-functioning EM team contributes to the overall efficiency and quality of healthcare [9, 10, 12]. By optimizing the initial evaluation and management of acutely ill patients, standardized training programs ensure a safe and secure management of acute medical and surgical emergencies. In the setting of increasing patient volumes and complexity, the benefits of an established training program were a common motivating factor in the specialization of the countries interviewed (see Appendix A).

The establishment of EM as an independent medical specialty has also served as a catalyst for further growth, including expanded research and the development of EM subspecialties [15]. As a result of this growth, EM has taken a central role in the creation of emergency response teams, providing a trained, responsive workforce capable of mobilizing for mass casualty incidents, pandemics, and natural disasters. This specialized preparedness enhances the resilience of the healthcare system, equipping it to handle patient surges during public health crises. The World Health Organization (WHO) recognized this in their 2019 Assembly, stating that “robust emergency (…) services are at the foundation of national health systems’ ability to respond effectively to emergency events” [16]. Thus, EM is essential to enhancing the resilience and sustainability of healthcare systems worldwide. By ensuring that emergency care is managed by specialized professionals dedicated to emergency care, patients ultimately benefit [17].

Acknowledgement of the impact of EM specialty recognition on the recruitment and retention of EM physicians was also a common theme among those countries reviewed [18]. Streamlining the training structure, and reducing the time spent training outside of EM, promotes the specialty as a primary career pathway, which could help address staffing shortages, reduce dependency on staff from other specialties, and alleviate trainer fatigue from repeatedly training professionals who eventually leave the field [19]. As the specialty of EM becomes a global norm, attracting international EM physicians who may be deterred by the lack of EM specialty recognition was also a shared motivating factor. Standardized training such as through the European Board Examination in Emergency Medicine (EBEEM) facilitates professional mobility within the European Union [20, 21].

### Benefits for well-being of healthcare professionals

A recent survey of 18 experts from six European countries including Germany identified that recognizing the specialty of EM is conceived as a key factor for improving working conditions [22]. This distinction enhances professional fulfilment by addressing several of the WHO psychosocial risks for poor mental health at work, including lack of control over one’s work environment, role ambiguity, and career stagnation and uncertainty [23]. This recognition fosters a sense of professional identity and pride among EM practitioners, contributing to greater job satisfaction and morale.

Formal recognition of the specialty of EM helps ensure the allocation of essential resources, including appropriate staffing levels, mental health support, and career development opportunities, thereby promoting a more supportive work environment. It also empowers EM professionals to advocate for improved policies, including fair scheduling and robust safety protocols, which further enhance their well-being and job satisfaction.

## Critiques of recognizing emergency medicine as a specialty

While recognizing EM as a specialty has many benefits, there are also potential drawbacks and challenges [24].

### Disadvantages for patient care and outcomes

With specialization of EM comes the potential of inappropriate diversion of resources from other key areas. Care in the ED cannot and should not replace primary care services. While investing in EM as a specialty, it is critical to ensure that resources are not diverted from primary care and other essential health services. Additionally, increased focus on acute medical care, including management of stroke, trauma and cardiac events, could inadvertently deprioritize other groups.

Another risk of developing EM as an independent specialty is the potential for unrealistic expectations. In Europe, in countries where EM is still not a specialty (see Fig. 1), an argument often put forward by internal medicine, surgery, or anesthetics societies is the concern about the inadequate depth of knowledge of EM specialists with regard to medical patients, particularly those with multiple comorbidities [25]. While only an opinion, this demonstrates a lack of understanding of the actual work done in EDs worldwide. The primary goals of EPs are to treat time-sensitive conditions and connect patients with the appropriate resources for ongoing care. Furthermore, EPs determine if (and which) specialty evaluations are indicated and the timeframe for access, using their expertise to differentiate medical cases from surgical ones as well as to manage those cases requiring multiple specialist evaluations in parallel [26]. Such interdisciplinary care is far more challenging in a system that triages patients directly to medical or surgical subspecialty teams upon presentation to the ED [27].

Finally, with pressure to establish a diagnosis or to facilitate outpatient workups, EPs will often go beyond treating the acute medical condition. To minimize overcrowding, over-testing, and increased costs, medical, legal and legislative bodies must support EPs in deferring workup of non-acute conditions to outpatient primary care or specialty teams.

### Disadvantages for the healthcare system

The financial and institutional support for creating specialized training programs and facilities, and for fulfilling staffing requirements, is often provided as a barrier to EM specialization [28]. This initial investment may be inaccessible to some, particularly in rural and underserved areas, potentially increasing health disparities [29]. Nonetheless, implementing the standards of dedicated training, and requiring all centers to be staffed by certified physicians, will improve accountability of the healthcare system to populations, regardless of their place of residence.

The development of new processes to ensure integration of EM services into the broader healthcare continuum requires interdisciplinary collaboration, which itself may be challenged in the transition from staffing the ED with a variety of medical and surgical subspecialties to ED trained physicians. Clinical rotations for non-EM trainees in the ED not only provide valuable medical training but also serve as a basis for ongoing collaboration.

From a healthcare perspective, there are often unrealistic expectations and over-dependence on emergency care. Oftentimes this overreliance on EM is seen as a solution to disguise the under-support of social systems, which are the stepping stone to addressing issues such as access to medical care, poverty, homelessness, and addiction. To appropriately respond to these societal needs, broad collaboration is necessary between communities, legislatures, social systems, and EM. Recognizing EM as an independent specialty can further enhance this collaboration.

### Disadvantages for well-being of healthcare professionals

While the recognition of EM as a specialty offers professional benefits, it also has the potential to negatively impact provider well-being. The desirability, or lack thereof, of work in the ED may be seen as a disadvantage to EM specialization [28]. Reduced working hours (in comparison with other specialties), increased financial compensation, and improved shift structures have all been suggested as methods for improvement. Staffing the ED with various medical and surgical subspecialists increases the number of physicians who are qualified to work in the ED and may therefore enable broader distribution of less desirable shifts. Such a model may also allow for reduced working hours and increased flexibility to change one’s clinical practice as well as to pursue non-clinical activities such as teaching, research, quality management, administration, and non-professional measures of maintaining one’s well-being. Regardless of specialty status, sufficient awareness, training, and support of the well-being of healthcare professionals are critical to maintaining a functional healthcare system.

## Strategies in transitioning to emergency medicine specialty recognition

For systems contemplating the recognition of EM as a specialty, several strategies can facilitate this transition. Firstly, *network *with other professionals to advocate for patients and providers. Building strong connections with established local or even international professionals and organizations can provide invaluable guidance, support and even funding, as seen in countries such as Romania, Belgium, Italy, and the Netherlands.

*Standardize training* in EM. Evidence-based, standardized, and interactive training is crucial to guaranteeing high-quality emergency care across diverse environments [20, 21, 30]. Standardized training also strengthens the credibility of EM as a discipline, highlighting its unique contributions to the broader healthcare system. Additionally, committing to ongoing improvement helps the specialty adapt to evolving practices and challenges, further solidifying its role in delivering the best patient outcomes possible.

**Fig. 3:**
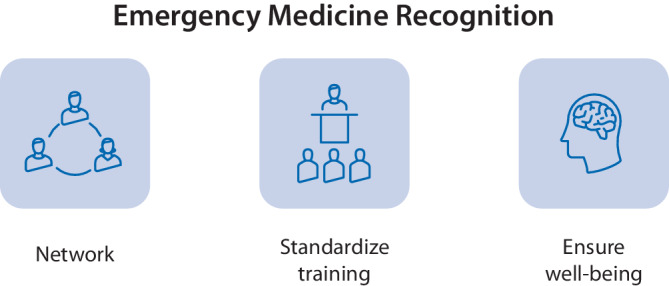
Strategies in transitioning to emergency medicine specialty recognition

Finally, *ensure the well-being* of the EM team. Professional well-being is of vital importance for longevity and retention [19, 31]. Developing strategies to manage stress, avoid burnout, and maintain work–life balance provides sustainability both for individuals and for the specialty as a whole (see Fig. 3).

## Conclusion

As representatives of the European Society for Emergency Medicine Well-being Working Group, we call for global specialty recognition as the next step to improving patient care, working conditions, and well-being in emergency medicine (EM). The formalization of EM as a specialty is the cornerstone of empowerment for EM professionals. It strengthens healthcare systems by promoting responsive, high-quality care for patients and cultivating a healthier, more sustainable work environment for emergency physicians.

## Supplementary Information

Appendix A: Review of the recognition process and impact observed in eight different countries

## Declarations

For this article no studies with human participants or animals were performed by any of the authors. All studies mentioned were in accordance with the ethical standards indicated in each case.
