# Supplementary material for: A team without a name: emergency medicine recognition and its impact on working conditions and well-being
Source: Med Klin Intensivmed Notfmed. 2025 May 2;120(6):481–6. doi: 10.1007/s00063-025-01275-8 (PMC12411600; doi:10.1007/s00063-025-01275-8)
Supplement: Supplementary file 1 — Appendix A: Review of the recognition process and impact observed in eight different countries [file 63_2025_1275_MOESM1_ESM.pdf]

Appendix A: **Review of the recognition process and impact in eight different countries**

Country (sources)	When was SR achieved?	Why was SR achieved?	How was SR achieved?	Immediate impact of SR	Impact 5 years after SR	Current state of the workforce
**USA (1-3)**	1979	b	i	m	m	t; v
**Romania (4-6)**	1999*	a; b; f	h	l	o; p; r	t; u; w; y
**Belgium (7-11)**	2005	a; b	g	j; k; l	o; p	t; u; w; y
**Italy (12-16)**	2009	a; b; d; f	g	l	o; q	t; u
**Israel (17-21)**	2012**	b; c; d; f	g	m	o	u; v
**Netherlands (22-26)**	2023	a; d	g	l; n	m; q; s	t; x
**Portugal (27-30)**	2024	b; d; e	g; i	m	-	-
**Spain (31-32)**	2024	b;d	i	k; l	-	-

SR: specialty recognition
*1993: 3-year program; 1999: 5-year program; ** 1999: subspecialty; 2012: full specialty

Why was specialty recognition achieved?	Impact 5 years after specialty recognition
a) growing complexity of patients<br>b) increased need for specialized/standardized/qualitative EM care and augmented efficiency<br>c) uniqueness of work<br>d) need for standardization of training<br>e) derived benefits (management, research, public health, disaster medicine)<br>f) social aspects (role, socio-political opportunity)	m) training opportunities<br>o) improvement of working conditions (resources, staffing, training)<br>p) high levels of stress<br>q) recognition of EM professionals' contribution to patient care and increased patient satisfaction<br>r) formal legislative recognition<br>s) nationwide standardization of protocols and guidelines
**How was specialty recognition achieved?**<br>g) concerted efforts by pioneering EM physicians, alongside government and national professional societies<br>h) concerted efforts by pioneering physicians, alongside international support<br>i) Collaboration and advocacy of community physicians in the setting of increasing demand for hospital-based care<br><br>**Immediate impact of specialty recognition**<br>j) job security<br>k) professional respect<br>l) improved quality of care and support systems<br>m) training opportunities<br>n) diminished workload for other specialties	**Current state of the workforce**<br>t) high demand and pressure (due to patient volume and complexity, staffing shortages, increased boarding time, shortcomings of primary care, financial incentives)<br>u) burnout<br>v) standards of training<br>w) ongoing need for addressing overall wellbeing of EPs<br>x) ongoing need for interdisciplinary collaboration<br>y) lack of research engagement

Overview of unique contributing factors and circumstances

In the **USA**, EM is well-established, with over 45 years of recognition, 1500 new EPs yearly and 16 different subspecialties. The rising involvement of for-profit entities in ED management creates significant conflicts in patient care.	In **Israel**, the 1991 Gulf war was a catalyst to the establishment of the Israeli Association of Emergency Medicine, promoting recognition. Today, the National Israeli Course of Emergency Medicine helps maintain a high standard of training.
In **Romania**, the 1989 fall of communism created an opportunity for EM development in both pre- and in- hospital settings.	In the **Netherlands**, EM development was a collaborative effort based on international models. Recognition bought standardization of protocols and guidelines and increased patients' trust in the system.
In **Belgium**, EM recognition was made possible through the work of pioneering EPs and brought a significant improvement in working conditions. Burnout remains a major issue	**Portugal** achieved EM recognition in September 2024. They plan to start training EM specialists in 2025.
In **Italy**, the ED plays a crucial role in filling gaps in the primary care and social support systems.	In **Spain**, recent recognition brought an immediate boost in physicians' sense of wellbeing.

References:

1. Suter RE. Emergency medicine in the United States: a systemic review. World J Emerg Med. 2012;3(1):5–10.
2. Merritt AK. The Rise of Emergency Medicine in the Sixties: Paving a New Entrance to the House of Medicine. J Hist Med Allied Sci. 2014 Apr 1;69(2):251–93.
3. Huecker MR, Shreffler J, Platt M, O'Brien D, Stanton R, Mulligan T, et al. Emergency Medicine History and Expansion into the Future: A Narrative Review. West J Emerg Med. 2022 May;23(3):418–23.
4. Arafat R. Evolutia si organizarea Sistemului integrat de urgenta din Romania [Internet]. 2019 [cited 2024 Nov 26]. Available from: https://media.dcnews.ro/other/202204/evolutia-si-organizarea-sistemului-integrat-de-urgenta-din-romania_nancycaroline_26218800.pdf
5. Puticiu M, Grecu MB, Rotaru LT, Butoi MA, Vancu G, Corlade-Andrei M, et al. Exploring Burnout, Work Addiction, and Stress-Related Growth among Prehospital Emergency Personnel. Behav Sci Basel Switz. 2024 Sep 22;14(9):851.
6. Mureşan EM, Golea A, Bolboacă SD, Perju-Dumbravă L. Feasibility of a pilot study on point-of-care biomarkers in spontaneous intracerebral hemorrhage in an emergency setting. Med Pharm Rep. 2021 Jul;94(3):307–17.
7. Van den Heede K, Ghesquiere A, Misplon S, Quentin W, Loon CV. Organisation and payment of emergency care services in Belgium: current situation and options for reform. 2016;
8. Stroobants J, Arafat R, Leach R, Halpern P, Golea A, Heyworth J, et al. Cooperation in emergency medicine in Europe: the bright side of the medal. Eur J Emerg Med Off J Eur Soc Emerg Med. 2018 Feb;25(1):1–2.
9. Somville F. Burnout among emergency physicians : from detection to prevention [Internet]. University of Antwerp; 2024 [cited 2024 Sep 15]. Available from: https://hdl.handle.net/10067/2052510151162165141
10. Somville F, Van der Mieren G, De Cauwer H, Van Bogaert P, Franck E. Burnout, stress and Type D personality amongst hospital/emergency physicians. Int Arch Occup Environ Health. 2022 Mar 1;95(2):389–98.
11. Somville F, Van Bogaert P, Wellens B, De Cauwer H, Franck E. Work stress and burnout among emergency physicians: a systematic review of last 10 years of research. Acta Clin Belg. 2024 Jan 2;79(1):52–61.
12. DECRETO DEL PRESIDENTE DELLA REPUBBLICA 10 dicembre 1997, n. 484 - Normattiva [Internet]. [cited 2024 Dec 11]. Available from: https://www.normattiva.it/uri-res/N2Ls?urn:nir:presidente.repubblica:decreto:1997-12-10;484

13. Ministero della Salute [Internet]. 1998 [cited 2024 Dec 11]. Available from: https://www.quotidianosanita.it/allegati/allegato1662036999.pdf
14. Coen D, Casagranda I, Cavazza M, Cervellin G, Ghiadoni L, Lerza R. Facing the Emergency Department crisis in Italy. Emerg Care J [Internet]. 2021 Dec 20 [cited 2024 Dec 11];17(4). Available from: https://www.pagepressjournals.org/ecj/article/view/10331
15. Piazza I, Barcella B, Cascio M, Group C. A national survey of Italian emergency medicine residents: it's time to stay and play. Emerg Care J [Internet]. 2024 Jun 18 [cited 2024 Dec 11];20(2). Available from: https://www.pagepressjournals.org/ecj/article/view/12671
16. Cascio M, Barcella B, Zaccaria G, Piazza I, Group C. Italian Emergency Medicine residents' perspectives. Emerg Care J [Internet]. 2022 Sep 27 [cited 2024 Dec 11];18(3). Available from: https://www.pagepressjournals.org/ecj/article/view/10870
17. Halpern P, Waisman Y, Steiner IP. Development of the specialty of emergency medicine in Israel: comparison with the UK and US models. Emerg Med J EMJ. 2004 Sep;21(5):533–6.
18. Waisman Y, Amir L, Or J. Emergency medicine in Israel: state of the art. Ann Emerg Med. 1995 Nov;26(5):640–2.
19. Drescher MJ, Aharonson-Daniel L, Savitsky B, Leibman J, Peleg K. A study of the workforce in Emergency Medicine in Israel: 2003. J Emerg Med. 2007 Nov;33(4):433–7.
20. Drescher MJ, Wimpfheimer Z, Darawsha A, Sullivan R, Goral A, Aharonson-Daniel L. A study of the workforce in emergency medicine in Israel 2012: what has changed in the last decade? Int J Emerg Med. 2015 Dec;8(1):47.
21. Shopen N, Tshuva R, Drescher MJ, Glatstein M, Cohen N, Coral R, et al. The Evolution of Board-Certified Emergency Physicians and Staffing of Emergency Departments in Israel. West J Emerg Med Integrating Emerg Care Popul Health [Internet]. 2024 Jun 14 [cited 2024 Nov 24];25(4). Available from: https://escholarship.org/uc/item/1m5476t8
22. Emergency medicine in the Netherlands - Holmes - 2010 - Emergency Medicine Australasia - Wiley Online Library [Internet]. [cited 2024 Oct 12]. Available from: https://onlinelibrary.wiley.com/doi/abs/10.1111/j.1742-6723.2009.01259.x
23. Windget P. Eusem - Breaking news: Emergency Medicine Takes a New Turn in the Netherlands [Internet]. [cited 2024 Dec 11]. Available from: https://eusem.org/news/919-breaking-news-emergency-medicine-takes-a-new-turn-in-the-netherlands
24. Kleijne I. Spoedeisende geneeskunde alsnog erkend als specialisme [Internet]. 2023 [cited 2024 Dec 11]. Available from: https://www.medischcontact.nl/actueel/laatste-nieuws/nieuwsartikel/spoedeisende-geneeskunde-alsnog-erkend-als-specialisme
25. Kleijne I. SEH-arts erkend als medisch specialist – wat betekent dat? [Internet]. 2023 [cited 2024 Dec 11]. Available from: https://www.medischcontact.nl/actueel/laatste-nieuws/artikel/seh-arts-erkend-als-medisch-specialist-wat-betekent-dat
26. Ikkersheim DE, van de Pas H. Improving the quality of emergency medicine care by developing a quality requirement framework: a study from The Netherlands. Int J Emerg Med. 2012 May 23;5(1):20.
27. Torres RRG. Outcomes of having a nationwide specialty in Emergency Medicine. 2020 [cited 2024 Nov 29]; Available from: https://repositorio-aberto.up.pt/bitstream/10216/128504/2/412107.pdf
28. The Portuguese Medical Association approves Emergency Medicine specialty [Internet]. 2024 [cited 2024 Dec 11]. Available from: https://www.portugalpulse.com/the-portuguese-medical-association-approves-emergency-medicine-specialty/
29. Kaur D. Eusem - Congratulations to Portugal on the Approval of the Specialty of Emergency Medicine [Internet]. [cited 2024 Dec 11]. Available from: https://eusem.org/news/974-congratulations-to-portugal-on-the-approval-of-the-specialty-of-emergency-medicine
30. UHPR National Secretariat. National Report of Portugal: Universal Health and Preparedness Review (UHPR) Pilot [Internet]. Lisbon; 2022 [cited 2024 Dec 11]. Available from: https://apps.who.int/gb/MSPI/pdf_files/2023/12/Item2_19-12.pdf
31. Ministerio de la Presidencia, Justicia y Relaciones con las Cortes. Real Decreto 610/2024, de 2 de julio, por el que se establece el título de Médica/o Especialista en Medicina de Urgencias y Emergencias y se actualizan diversos aspectos en la formación del título de Médica/o Especialista en Medicina Familiar y Comunitaria [Internet]. Sect. 1, Real Decreto 610/2024 Jul 3, 2024 p. 82078–89. Available from: https://www.boe.es/eli/es/rd/2024/07/02/610
32. Redacción. Médicos y Pacientes - Portal informativo de la Organización Médica Colegial. 2024 [cited 2024 Nov 24]. La creación de la especialidad de Urgencias y Emergencias, un paso adelante. Available from: https://www.medicosypacientes.com/articulo/la-creacion-de-la-especialidad-de-urgencias-y-emergencias-un-paso-adelante/
